# Chirality at nanoscale for bioscience

**DOI:** 10.1039/d1sc06378b

**Published:** 2022-02-08

**Authors:** Maozhong Sun, Xiuxiu Wang, Xiao Guo, Liguang Xu, Hua Kuang, Chuanlai Xu

**Affiliations:** International Joint Research Laboratory for Biointerface and Biodetection, State Key Lab of Food Science and Technology, School of Food Science and Technology, Jiangnan University Wuxi Jiangsu 214122 People's Republic of China smz@jiangnan.edu.cn xcl@jiangnan.edu.cn

## Abstract

In the rapidly expanding fields of nanoscience and nanotechnology, there is considerable interest in chiral nanomaterials, which are endowed with unusually strong circular dichroism. In this review, we summarize the principles of organization underlying chiral nanomaterials and generalize the recent advances in the main strategies used to fabricate these nanoparticles for bioscience applications. The creation of chirality from nanoscale building blocks has been investigated both experimentally and theoretically, and the tunability of chirality using external fields, such as light and magnetic fields, has allowed the optical activity of these materials to be controlled and their properties understood. Therefore, the specific recognition and potential applications of chiral materials in bioscience are discussed. The effects of the chirality of nanostructures on biological systems have been exploited to sense and cut molecules, for therapeutic applications, and so on. In the final part of this review, we examine the future perspectives for chiral nanomaterials in bioscience and the challenges posed by them.

## Abstract 2

In this review, we summarize the principles of fabrication on chiral nanomaterials and generalize the recent achievements for the bioscience applications.

## Graphical abstract

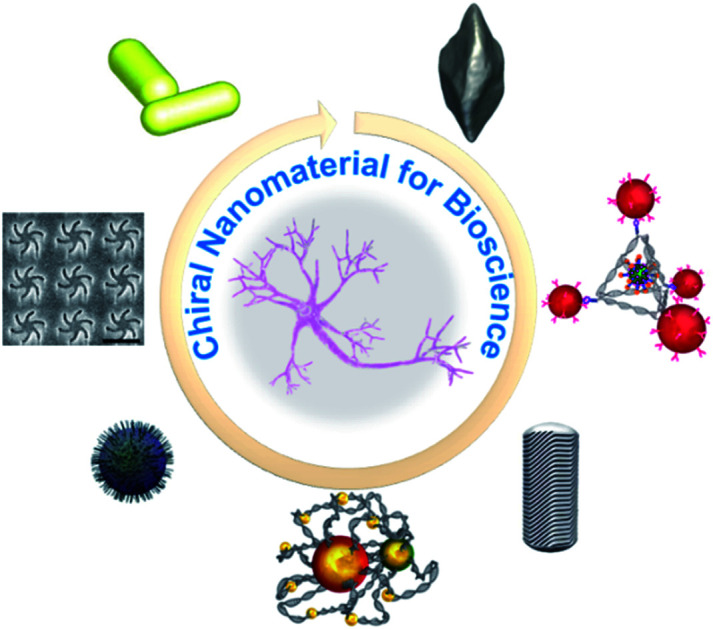

## 1. Introduction

Chirality is a property whereby a chiral object is not superimposable upon its mirror image. A substance and its nonsuperimposable mirror counterpart are called ‘enantiomers’, and have similar physical properties but cause linearly polarized (LP) light to rotate in different directions. Dextrorotatory (d) enantiomers rotate LP light in a right-handed or clockwise direction, whereas the levorotatory (l) enantiomers rotate LP light in a left-handed or counterclockwise direction. This phenomenon is called ‘optical activity’, and can be described by the conventional spectroscopic technique of circular dichroism (CD), which measures the different absorption of left and right circularly polarized light (CPL) by chiral materials. The anisotropy factor (*g*-factor) is also used to quantitatively describe the optical properties of chiral materials. Chirality is ubiquitous in nature, occurring in molecules, proteins, nucleic acids, and biological morphologies.^1^ The preferences of many biomolecules for specific enantiomers are universal in living bodies. One enantiomer of a chiral material may be a building block for a life activity, whereas the other one is ineffective or even toxic. Chirality is also a fundamental property of molecular recognition during metabolism. The exploitation of chirality at the nanoscale is regarded as one of the most promising areas in nanomaterial research today. The associations between the morphology, scale, charge, and chirality of biomolecules and those of chiral nanomaterials are currently drawing considerable attention in various research fields. Unlike achiral materials, chiral nanomaterials have excellent optical properties, which allow them to be used as a nanotechnological tool for the detection of biological compound.^2^

The dissymmetric arrangement of the components of chiral materials, together with the influence of various extraneous fields, play a crucial role in controlling their properties.^3^ In achiral structures, the arrangements of these nanoscale building blocks lack ‘handedness’ features, limiting their utility in recognition in biosystems. Interestingly, chiral structures can be prepared spontaneously with various approaches, according to human design. The unique spatial arrangements of chiral nanomaterials allow greater control of the collective interactions between the different building blocks of the materials and their interactions with other molecules. Understanding the construction of these nanoscale building blocks into unique chiral spatial architectures allows the orientations and chiral optical properties of these architectures to be controlled. Furthermore, the simulation of the chiral morphologies of nonliving systems, assembled from inorganic metal nanoparticles (NPs), should facilitate the analysis of natural structures in biology.

**Fig. 1:**
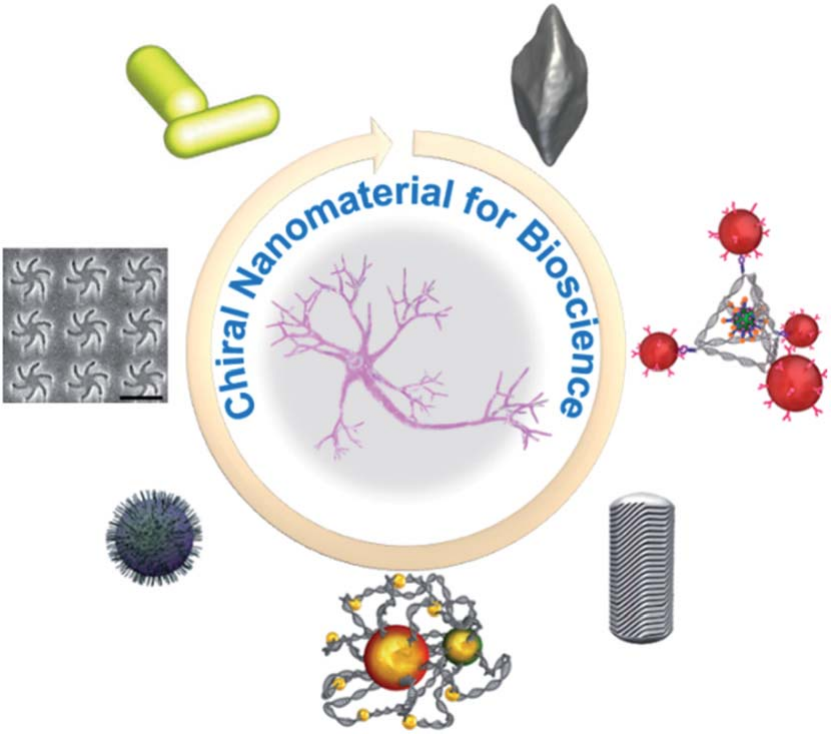
Scheme of chiral nanomaterials for bioscience: chiral configurations of Au NR dimer–BSA complexes. Adapted with permission from ref. 82. Copyright © 2019 American Association for the Advancement of Science. Electron microscopy of chiral films. Adapted with permission from ref. 71. © 2018 American Chemical Society. Synthesis of chiral Fe_*x*_Cu_*y*_Se NPs. Adapted with permission from ref. 102. Copyright © 2020 Wiley-VCH Verlag GmbH & Co. KGaA, Weinheim. Chiral self-assembly stimulate the differentiation of neural stem cells. Adapted with permission from ref. 107. Copyright © 2021 Springer Nature. Micelle-directed chiral gold nanocrystals. Adapted with permission from ref. 18. Copyright © 2020 American Association for the Advancement of Science. Chiral tetrahedron for clearance of senescent cells. Adapted with permission from ref. 98. Copyright © 2020 WILEY-VCH Verlag GmbH & Co. KGaA, Weinheim. Morphology of chiral bipyramids nanocrystals. Adapted with permission from ref. 91. Copyright © 2021 American Association for the Advancement of Science.

The excellent characteristics of chiral nanostructures mean that they have been extensively studied and have practical value in the fields of modern nanophotonics, including quantum electrodynamics, ultrasensitive biological detection, antibacterial processes, precise chemical analyses, and understanding the chiral effects in living systems.^4–7^ Several approaches have been developed by scientists around the world to precisely prepare chiral nanomaterials. These can be classified into three types: chiral assemblies of achiral building blocks; NPs with intrinsic chiral morphologies; and achiral NPs covered with chiral ligands. Their extraordinary applications have been investigated.^1,6,8–10^ In this review, we focus on the fundamental construction of chiral nanomaterials, including template-assisted chiral nanomaterials, chiral nanoassemblies, chiral quantum dots, chiral nanofilms, plasmonic chirality at the nanoscale, and chiral nanocrystals, and their applications in bioscience (Fig. 1). To enhance and customize the chiral optical responses of chiral structures, their morphologies and spatial conformations can be controlled by different stimuli.^11–14^ Engineering the tunable optical, electronic, magnetic, and catalytic properties of chiral nanomaterials depends on the precise control of the NP size, composition, surface chemistry, and spatial distribution.

## 2. Fabrication of chiral nanomaterials and their applications in bioscience

### 2.1 Template-assisted chiral nanomaterials

**Fig. 2:**
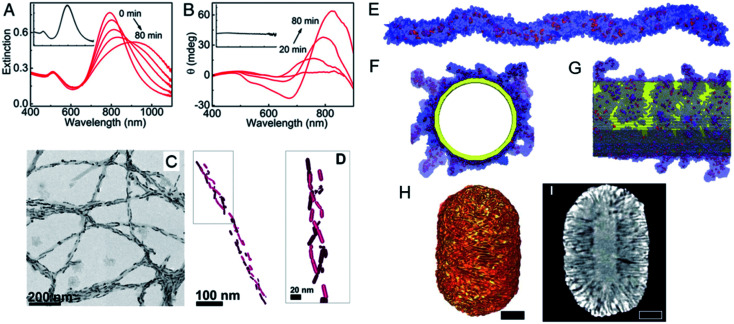
(A) Extinction, (B) CD spectral changes, (C) TEM images and (D) Cryo-TEM tomography reconstruction image of Au NRs after the addition of α-synuclein fibrils. Adapted with permission from ref. 16. Copyright © 2018 National Academy of Sciences. (E–G) MD simulations of chiral, worm-like (*R*)-BINOL-CTAC micelle before and after adsorbed onto a gold nanorod. (H and I) Tomography reconstruction a of Au NR grown in an (*R*)-BINAMINE-surfactant mixture. Scale bar, 100 nm Adapted with permission from ref. 18. Copyright © 2020 American Association for the Advancement of Science.

A variety of strategies are used to fabricate chiral nanostructures.^15^ The simplest route is based on a chiral template, in which the translation of the conformation of the template into building blocks allows the researcher to guide the self-assembly or synthesis of template-based nanoscale materials. A patterned physical template, such as a gel, micelle, polymer, mesoporous silica, or supramolecular fiber, provides a rigid geometric structure. After its assembly, the building blocks are arranged around or along the well-defined shape or structure of the template. Luis M. Liz-Marzan's group reported the use of amyloid fibrils or chiral fibers as scaffolds for nanorod (NR) assembly.^16,17^ With this method, the dispersion of heterogeneous NRs was guided into a chiral conformation based on the template, and a strong surface plasmon-mediated CD (SP-CD) signal was first obtained with nonspherical metal NPs. Chiral amyloid was used as the fiber backbone template upon which the helical morphology of the NRs was constructed. The assembled NRs showed strong plasmonic CD intensity and a chiral arrangement in solution after the addition of the fibers (Fig. 2A–D). The signal of the assembly in the plasmon resonance region had low background interference and interacted specifically with helical protein fibrils. The method developed was used to identify these fibrils in human brain homogenates from patients with Parkinson's disease, confirming its potential application in the detection of clinical neurodegenerative diseases. As well as the nano-assembly of achiral NPs, single NPs with chiral configurations can also be constructed with template mediation. Chiral micelles of 1,1′-bi(2-naphthol) (BINOL), a cosurfactant, have recently been used as a template for the seeded growth of anisotropic gold nanocrystals. The adsorption of the micelles to gold NRs induced the assembly of a surfactant (cetyltrimethylammonium chloride [CTAC]) into chiral molecules that formed quasihelical patterns (Fig. 2E–G). These directed the diffusion of the gold-containing micellar aggregates and induced the formation of wrinkles on the NR surfaces (Fig. 2H and I). Notably, the optical activity of the NRs could be modulated by varying their aspect ratio, and high anisotropy factors of about 0.20 were obtained from 500 to >1350 nm, which are the highest values reported for colloidal plasmonic NPs in the biological activity window of the near-infrared region.^18^ A theoretical analysis revealed that the chirality originated from the coiling morphology in opposite directions.

DNA origami is a versatile and robust template for the construction of chiral nanomaterials.^19–22^ The DNA structures can be tailored to have well-defined spatial configurations, from which plasmonic chiral structures can be constructed through self-assembly.^23^ DNA can be used as the construction templates upon which plasmonic NPs are organized into chiral conformations through the ingenious design of DNA origami.^24^ The different handedness in space and plasmon coupling can be programmably controlled in highly ordered ways. The research group of Na Liu and Tim Liedl first designed a reconfigurable DNA origami template that produced a chiral arrangement of gold NRs with a strong CD signal.^25^ The chirality of these assemblies arises from both the structural chirality and plasmonic chirality. Moreover, in different forms of chiral micelles or gels, the handedness or the morphology of the template (DNA origami) can be manipulated as desired, to provide infinite potential applications. Therefore, the optical response obtained can generate the ideal symmetrical spectrum. The interaction between light and matter at the nanoscale can also be exploited by walking NRs stepwise, both directionally and progressively, on DNA origami, using DNA as the fuel.^26^ With this method, different plasmonic stereoisomers with various chiral centers have been constructed.^27,28^ Importantly, the strong CD signal was used to sensitively and selectively detect a specific target sequence, with which the cross angle of the assembly could be immobilized. Defining the state of the plasmonic configuration allowed a distinct optical response to be generated, with great potential utility in biodetection.^29^ Tim Liedl's group detected and quantified an RNA sequence of *Hepatitis C virus* (HCV), with a limit of detection (LOD) of 100 pM, which paved the way to use DNA origami and chiral nanomaterials as rapid detection reagents for pathogenic nucleic acids.^30^

**Fig. 3:**
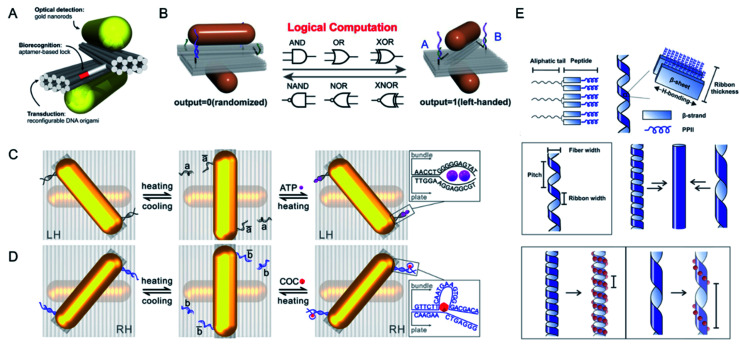
(A) Schematics of the chiral plasmonic nanosensor through DNA origami. The DNA aptamer-based lock is used as a biorecognition component incorporated into reconfigurable DNA origami structure. Adapted with permission from ref. 29. Copyright © 2018 American Chemical Society. (B) Schematics of DNA-based plasmonic logic gate. Adapted with permission from ref. 32. Copyright © 2020 Wiley-VCH Verlag GmbH & Co. KGaA, Weinheim. (C and D) Schematic of the thermal and split ATP aptamer or cocaine aptamer-target regulations of the plasmonic system. Adapted with permission from ref. 31. © 2018 American Chemical Society. (E) Schematics of helical ribbon assembly with C_18_-(PEP^M-ox^_Au_)_2_ and demonstrating the helical ribbon morphology may affect the helical pitch of gold NP single helices. Adapted with permission from ref. 38. © 2017 American Chemical Society.

Other external inputs was integrated with two kinds of aptamers and a logical gate can be designed based on this platform for biological applications (Fig. 3A–D).^31^ It was functionalized with both adenosine triphosphate (ATP) and cocaine aptamers, which can both be activated thermally. By exploiting the high specificity and selectivity of aptamers, more-dynamic plasmonic probes for various molecular targets can be constructed with DNA origami.^27,32–35^

Soft templates other than DNA molecules, such as peptides, have also been used in the assembly of helically arranged gold NPs or nanowires (Fig. 3E).^36,37^ A family of helical gold NP assemblies was prepared with C_18_-(PEP^M-ox^_Au_)_2_ (PEP^M-ox^_Au_ = AYSSGAPPM^ox^PPF) by Professor Nathaniel L. Rosi's group.^38–41^ The peptide-conjugated molecules contained an organic tail that directed their self-assembly and bound to gold NPs, which displayed strong plasmonic chiroptical activity, with *g*-factor values of up to 0.04. The morphology and chiroptical activity of the nanowire could be tuned effectively by changing the aliphatic tail length, the helical pitch and size, the shape, and the aspect ratio of the NPs. The positions of methionine and methionine sulfoxide within the peptide sequence could also be changed to control the size and aspect ratio of the NPs, potentially enhancing the chiroptical properties of the helices. The biomolecule templates-based way for the construction of chiral nanomaterials displayed higher biocompatibility than other methods, with consequent potential utility in living systems.

### 2.2 Chiral nano-assembly

**Fig. 4:**
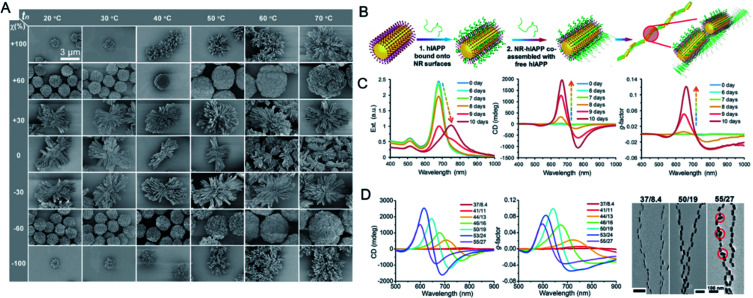
(A) Phase diagram and diversity of HOPs from chiral nanoplatelets. SEM images of Au-Cys particles assembled at different nucleation temperatures *t*_*n*_ and *χ* values. Adapted with permission from ref. 47. Copyright © 2020 American Association for the Advancement of Science. (B) Schematics of the assembly process of hIAPP monomers with NRs. (C) Extinction, CD, and *g*-factor spectra for the co-assembly process of hIAPPs with gold NRs respectively. (D) The influence of NR size and nanohelix pitch on experimentally observed and computationally derived *g*-factors. Adapted with permission from ref. 48. Copyright © 2021 American Association for the Advancement of Science.

Like the template-assisted methods, self-assembly is one of the commonly used approaches to the fabrication of chiral systems. In this section, we focus on the self-assembly processes that are mediated by small ligands attached on the surfaces of building blocks. Under different driving forces, isotropic NPs are directed into a series of discrete uniform patterns through self-assembly, which by definition, proceeds without human intervention.^42,43^ Professor Kotov's laboratory reported the fabrication by self-assembly of chiral inorganic particles with uniform enantiomeric configurations. They transferred nanoscale chirality to complex structures, driven by CPL or chiral amino acids. Strong chiroptical activity was obtained by tuning the noble metal thiolates and surface ligands. The chiral nanoribbons adopted twisted orientations of different handedness after racemic CdTe NPs were illuminated with CPL, resulting in an enantiomeric excess (e.e.) of 30%. This confirmed that CPL can be used as ‘a template’ for the self-assembly of enantioselective chiral NPs.^44^ Chiral self-sorting was also observed in cysteine (Cys)-stabilized CdTe NPs in methanol, and was attributed to a thermodynamic preference for homochiral NP assemblies. The e.e. of the final helical shape of the assembled structure was ≥98%, and the *g*-factor was 0.01, more than two orders of magnitude higher than that of the NPs.^45^ To further modulate chiral optical activities at the nanoscale in real time, chiromagnetic Co_3_O_4_ NPs with chirally distorted crystal lattices were synthesized.^46^ The chiroptical effects of the paramagnetic NPs in both dispersions and gels were 10 times stronger than those of nonparamagnetic NPs. The transparency of the Co_3_O_4_ gels to CPL was tunable under a magnetic field. The chirality-induced assembly of nanostructures has also been investigated. The use of noble-metal thiolates of Au–Cys allowed the formation of hierarchically organized particles (HOPs), in which the complexity displayed was extraordinarily higher than that in their biological counterparts or other complex NPs (Fig. 4A).^47^ The assembly pathways were dependent on the symmetry of the NPs rather than on their size, which indicated chirality-dependent assembly restrictions. The chiral configuration of the HOPs originated from the staggered arrangement of twisted nanoribbons. Moreover, the chiroptical properties of the HOPs could be tuned by doping them with coinage metals. Based on this report, the chirality of NPs should be considered to play a fundamental role in assembly pathways and warrants further research.

By mimicking the long-range organization of chiral molecules in liquid crystals, high optical asymmetry at the nanoscale level was achieved with the assembly of gold NRs into long helical chain-like NRs with an end-to-end orientation after they were conjugated to human islet amyloid polypeptides (hIAPPs) (Fig. 4B). After they were co-assembled with the nanohelix peptides, the NRs assembled into a long-range chiral form, and the optical asymmetry *g*-factor of the assembly was more than 4600 times higher than those of the monomer. The chiral response could also be regulated by adjusting the NR size and the helix pitch (Fig. 4C and D). Based on this design, more chiral nanomaterials with high optical asymmetry can be produced. More importantly, rapid sensing and drug discovery protocols can be developed for complex biological environments based on the plasmonic CD spectrum or polarization rotation imaging of chiral assembly, which has low background interference.^48,49^

### 2.3 Chiral quantum dots (QDs)

Chiral QDs are a promising material for applications in biological sensing, photonics, and luminescent devices because they have readily tunable optical activities.^50^ Most chiral optically active QDs with an achiral core are prepared with chiral ligands.^51,52^

**Fig. 5:**
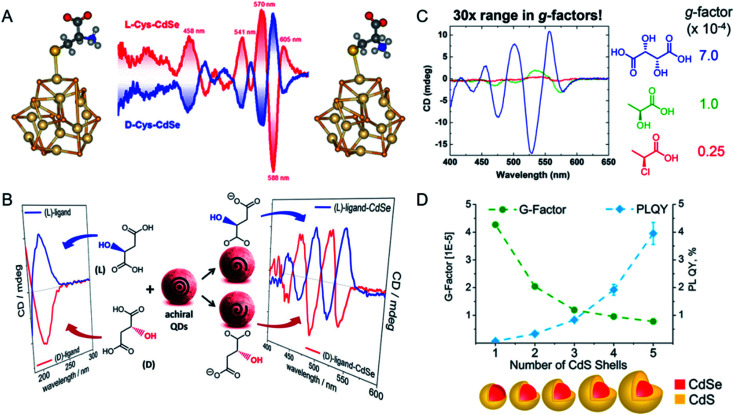
(A) Schematic of chiral CdSe QDs synthesized with ligands Cys. Adapted with permission from ref. 53. Copyright © 2013 American Chemical Society. (B) Schematic of chiral CdSe QDs synthesized with ligands thiol-free carboxylic acids. Adapted with permission from ref. 54. Copyright © 2017 American Chemical Society. (C) Enhance the dissymmetry factors in QDs by increasing the number of stereocenters on the ligand. Adapted with permission from ref. 55. Copyright © 2017 American Chemical Society. (D) Impact of shell thickness on chiral CdSe/CdS core–shell QDs. Adapted with permission from ref. 56. Copyright © 2017 American Chemical Society.

Professor Milan Balaz has studied the synthesis of chiral semiconductor NPs with different chiral ligands, and the relationships between the NP structures and ligands and their optical activity (Fig. 5A). Chiral CdSe QDs with l- or d-Cys ligands were prepared with a postsynthetic ligand exchange method.^53^ The optical activity induced showed size-dependent variations, and the origin of the signal was shown to be the orbital hybridization of the highest occupied CdSe molecule with a chiral ligand. Based on these results, further studies of ligand-induced chirality in QDs were undertaken with a thiol-free chiral carboxylic acid (malic or tartaric acid) as the capping ligand for the chiral CdSe QDs (Fig. 5B).^54^ Three oxygen-donor groups were shown to be necessary for the induction of chirality in CdSe, which in turn determined the CD signal. Stereoselective porphyrin-driven supramolecular nanoassemblies were also prepared on DNA templates. The photocatalytic activity of these nanostructures and the highly sensitive and selective spectroscopic detection were also studied. Experimental and theoretical calculations demonstrated that the attachment of chiral ligands to the surfaces of QDs generated the induced chirality through a bidentate interaction.

To further enhance the *g*-factors of chiral CdSe QDs, Vivian E. Ferry's group demonstrated that carboxylate-capped CdSe QDs showed more-intense CD signals than thiolate-modified CdSe QDs, with values reaching 7.0 × 10^−4^ (Fig. 5C).^55^ The origin of this chirality was examined from the splitting of the exciton by the interaction between the ligand and the QDs. The stereocenter of the ligand correlated positively with the *g*-factor of the chiral QDs. With these constructive advances, they confirmed that the chiroptical response of QDs can be tailored by the chemical structure of the chiral ligand. The hybridization of chiral ligands with the QD valence bands played a crucial role in their chirality, which was then influenced by the functional group or binding mode.

During the synthesis of QDs, a core–shell structure is one possible interesting design, which enhances the quantum yield of the photoluminescence. Based on this phenomenon, Professor Yurii K Gun'ko examined the synthesis of chiral core–shell QDs and the corresponding chiroptical activity (Fig. 5D). A series of CdS shells, with thicknesses ranging from 0.5 to 2 nm, were coated onto CdSe QDs.^56,57^ After ligand exchange with chiral Cys, chiral CdSe/CdS core–shell QDs were obtained. The chiroptical response was found to be reduced, whereas the photoluminescence of the QDs was enhanced, as the thickness of the CdS shell increased. These studies provided the foundations for the design of chiral fluorescent probes with QDs.

The chiral origin and optical properties of chiral semiconductor nanomaterials have been investigated by Professor Zhiyong Tang and colleagues, who have also explored the application of chiral materials to asymmetric catalysis, enantioselective separation, and chiral recognition. To explain the origins of and the changes in the CD responses in chiral semiconductor nanocrystals, in both the ultraviolet (UV) and visible regions, they fabricated chiral cysteine-molecule-stabilized CdSe quantum rods (QRs), with greatly enhanced CD responses. As the geometric aspect ratio of the QRs increased, the CD signal improved, and the optical activity of the colloidal semiconductor nanocrystals was induced by chiral organic ligands. The theory was explored with a nondegenerate coupled oscillator model, based on the different polarization of the excitonic transitions. Increasing the asymmetry of the QRs also increased the transition of the linearly polarized excitons, which caused stronger optical activity.^58,59^

Intrinsic chirality is also present in cinnabar mercury sulfide (HgS) nanocrystals, which have a clearly chiral geometric morphology.^60–63^ This chirality originates in the interplay between the chiral crystallographic lattice and the geometric handedness.

### 2.4 Chiral nanofilms

**Fig. 6:**
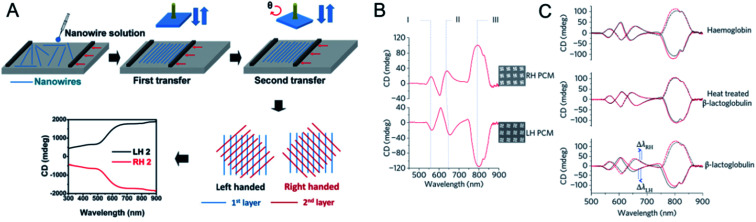
(A) Fabrication procedure and the optical activity of Au nanowire chiral ultrathin films. Adapted with permission from ref. 64. Copyright © 2017 Wiley-VCH Verlag GmbH & Co. KGaA, Weinheim. (B) CD spectra of PCMs immersed in distilled water. The three modes that show the largest sensitivity to changes in the local refractive index of the surrounding medium have been labelled I, II and III. (C) Influence of the adsorbed proteins haemoglobin, β-lactoglobulin and thermally denatured β-lactoglobulin on the CD spectra of the PCMs. Adapted with permission from ref. 67. Copyright © 2010 Macmillan Publishers Limited.

As well as single chiral NPs or chiral assemblies, two-dimensional chiral materials with distinct optical activities can also be produced. Zhiyong Tang's group fabricated large-area long-range ordered ultrathin chiral films using a bottom-up assembly method. Aligned one-dimensional ultrathin gold (Au) nanowires were prepared with the Langmuir–Schaeffer technique, and the subsequent layers were then rotated clockwise or anticlockwise at a predetermined angle to generate left- or right-handed ultrathin chiral films (Fig. 6A).^64^ The highest anisotropic factor (0.285) was obtained with three layers of Au nanowire (with an interlayer angle of 45°) in the wide 300–2000 nm wavelength range. Calculations demonstrated that the origin of the strong optical activity was the helical stacking of the layers of the anisotropic Au nanowire assembly. The universality of the method allowed its application to W_18_O_49_ and NiMoO_4_·*x*H_2_O. This technique was also used to fabricate left- or right-handed photonic crystals, and circularly polarized color reflection was observed with the clockwise or anticlockwise horizontal rotation of each layer.^65,66^

Kadodwala's group reported the generation of superchiral electromagnetic fields with gold gammadions of planar chiral metamaterials (PCM), which greatly enhanced the sensitivity of chiral molecules in chiroptical detection (Fig. 6B and C). The optical properties of left- or right-handed panels were used to determine the differences in chiral samples under left- and right-CPL.^67–71^ The interaction of chiral molecules with an electromagnetic field was enhanced by coupling them to the localized surface plasmon resonances (LSPRs) of gold gammadions. The shifts in the resonance wavelength induced by the adsorption of different chiral targets were detected for myoglobin, hemoglobin, and bovine serum albumin (BSA), which have high levels of α-helical secondary structure, and for β-lactoglobulin, outer membrane protein A, and concanavalin. Based on this method, the largest dissymmetries in the shifts of the LSPRs on adsorption of chiral layers was observed for biomolecules with many β-sheets, and was 10^6^ times higher than other optical polarimetry measurements.

Professor Shunai Che's group proposed a novel method of template-assisted hierarchical self-assembly for constructing highly ordered chiral mesoporous materials, in which a stack of chiral anionic organic templates induced chiral imprinting and amplification.^72^ They also generated mesoscopic structural materials *via* a chiral-molecule-induced pathway. Multilevel three-dimensional (3D) films (ZnO, silver, CuO, NiO, and so on), with hierarchical chirality from the atomic to the micron scales, were constructed using a symmetry-breaking agent, and their cooperative assembly effect was studied.^73–76^ Each film contained three levels of chirality, which were identified with structural characterization: (1) primary distorted nanoflakes/nanoplates with atomic crystal lattices; (2) secondary helical stacking of these nanoflakes to form nanoplates; and (3) tertiary micrometer-sized circinate aggregates of the chirally arranged nanoplates. Lattice defects were induced by the chiral center of the amino acid, which ultimately led to a circinate helical configuration. This research group suggested that the distorted crystalline structure and the helically aggregated nanoplates in these nanocrystals were smaller than Bohr's radius. The dynamic coulomb interaction in a dissymmetric field caused electron-transition-based optical activity. Moreover, scattering-based optical activity, CPL, and Raman optical activity were also generated with these nanocrystals, which could be applied to the chiral selection of amino acids. Very recently, arrays of chiral nanostructured Au films with high anisotropy were prepared for the discrimination of enantiomers with surface-enhanced Raman scattering (SERS) under linearly polarized or unpolarized light. They showed great versatility, detecting almost all enantiomers.^77^

### 2.5 Plasmonic chirality at the nanoscale

**Fig. 7:**
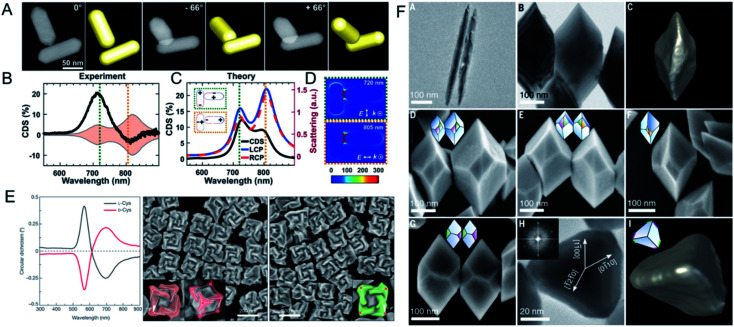
(A) HAADF-STEM tilt-series images of a chiral dimer in Au NR dimer-BSA complexes. (B) CDS spectra and (C) simulated scattering spectra of the chiral dimer. (D) The near-field enhancements calculation of the chiral NR dimer. Adapted with permission from ref. 82. Copyright © 2019 American Association for the Advancement of Science. (E) Different chiral 3D plasmonic helicoids obtained by cysteine chirality transfer. Adapted with permission from ref. 88. Copyright © 2018 Macmillan Publishers Limited, part of Springer Nature. (F) Morphology of chiral bipyramids nanocrystals. Adapted with permission from ref. 91. Copyright © 2021 American Association for the Advancement of Science.

Plasmonic nanostructures are one type of building block that can be used for chiral nanoprobe construction because they have enhanced electromagnetic near-fields. This method has received much attention because the nanostructures are easily accessible under mild synthesis conditions. As we mentioned above, chiral plasmonic nanostructures can be obtained with template-assisted or ligand-induced methods. Their intrinsic chiral morphology provides clear structural chirality.^78^ Moreover, like the structural chirality of NPs, the plasmon-coupled effect generates a strong plasmonic CD signal.^79^ A plasmonic NPs can induce the chirality of ligand amplification when it is closely located to the hotspot region, which is reportedly a promising approach for ultrasensitive biodetection.^80,81^ However, structural chirality and plasmon chirality are hard to distinguish in ensemble analyses. Professor Stephan Link investigated the origin of the optical activity of chiral molecules and nanomaterials, and identified the physical principles governing the interactions between plasmonic NPs and between these NPs and their chiral environments,^82^ with the goal of using plasmons, or light photons, to probe materials and initiate chemical reactions. Single-particle circular differential scattering spectroscopy (CDS) was used to differentiate the origin of chirality in the structural chirality of plasmonic aggregates or in the plasmon-coupled CD signal from that in gold NR–BSA complexes (Fig. 7A–D). The aggregation induced by NR–BSA produced a CDS signal, whereas no detectable signal was observed from single NR–BSA complexes or even single NRs with a BSA monolayer. The strong CDS signal from the aggregates was shown to originate from the intrinsic structural chirality, with a right-handed configuration. Electromagnetic hotspots enhanced the BSA-induced plasmon-coupled CD signal, which was confirmed with Raman spectroscopy. The conformation of the NR–BSA aggregates was visualized systematically using high-angle annular dark-field scanning transmission electron microscopy (HAADF-STEM), with tomographic reconstruction from different tilt angles and finite-difference time-domain (FDTD) simulations. The researchers found that the nonparallel dimer was chiral, with a strong CDS signal at 720 nm. Enhanced plasmon-coupled CD was also observed. The enhanced plasmon-coupled CD of a parallel achiral dimer was explained by the positions of the BSA molecules in the gaps between the NRs. The NR aggregates induced with NaCl, as a control, showed only structural chirality. From this study, the origin of chirality from complexes system can be determined from CDS measurements.

Dangyuan Lei's group fabricated a plasmonic chiral nanomaterial and investigated the chiral coupling through photonic spin–orbit interactions.^83^ With achiral nanostructures, they found that chiral cysteine molecules induced chiral optical activity on the surfaces of the NPs by inducing the formation of hydrogen-bond-connected helical networks of chemisorbed molecules on the NPs.^84^ This strong coulomb coupling and the local electric field of the plasmonic nanomaterial further enhanced the CD resonance. Notably, its intensity could be precisely defined and dynamically tuned by adjusting the solution temperature, pH, or the ions present.

The detailed bio-inspired chiral geometries of metallic nanostructures have been investigated by Ki Tae Nam's group.^85,86^ The chiral geometries of nanostructures are considered the key factors in enhancing their chiroptic responses (Fig. 7E).^87^ The biomolecule-based synthesis of 3D chiral plasmonic materials was examined in terms of peptide self-assembly and peptide interfaces with metallic materials. By investigating the mechanisms by which chiral geometries are involved in the formation of metallic nanostructures, these researchers used peptide-sequence-specific interactions with high-index surfaces to control the growth and optical properties of nanomaterials. A series of gold NPs with low-Miller-index surfaces were synthesized with a seed-mediated method.^88^ After these NPs had interacted with differently handed cysteine or cysteine-based peptides in the presence of Au^+^ ions, the low-index-plane exposed gold NPs evolved into high-index-plane NPs. The researchers demonstrated that the morphology and chiral optical properties of the NPs could be modulated by tuning the peptide sequence, amino acids, or gold seed. Because the growth rates of the chiral high-index planes of the NPs differed, the asymmetric evolution of the NPs ultimately led to the formation of helicoid morphologies. Based on this method, the strongest optical activity (with a *g*-factor of 0.2) was obtained when an octahedral seed was used.

### 2.6 Chiral nanocrystals

As well as plasmonic nanomaterials, chiral nanocrystals of inorganic materials have been prepared. Gil Markovich's group demonstrated that the interaction between chiral ligands, the spontaneous symmetry in lattice breaking, and the enantioselective nucleation of the growth chemistry during the crystallization process allowed the handedness of the nanocrystals to be controlled.^89,90^ Using thiolated chiral biomolecules as efficient reducing agents, chiral Te or Se NP precursors could be obtained. The handedness, chiral optical activity, and shape of the NPs could also be determined by the biomolecule used. The researchers found that the chiral configuration of the nanocrystals was formed by a unique self-assembly process, which was confirmed with a discrete dipole approximation simulation. The chirality originated not only from the chiral atomic lattice, but also from the mesoscale shape, in which the twisted ridges crossing diagonally between vertices defined the handedness.

Although chiral additives can determine a chiral shape, whether it is a necessary condition for a chiral crystal structure had not been tested. Therefore, Professor A. Paul Alivisatos's group investigated, in detail, the growth of chiral tellurium (Te) nanocrystals with either chiral or achiral ligands (Fig. 7F). They observed the chiral arrangement of a distinct structure of flat facets of thick trigonal–bipyramidal Te nanocrystals after chiral penicillamine was added to the reaction.^91^ However, the addition of a pure chiral ligand to the final reaction generated an e.e. (85%) of only one mirror, not 100%, indicating that the chiral ligand affected the nucleation and growth rates of the NPs. More importantly, when the achiral ligand mercaptopropionic acid was used instead of penicillamine, equivalent left- and right-handed nanocrystals were formed, demonstrating that chiral additives are not necessary for the formation of a chiral shape. Further studies with aberration-corrected HAADF-STEM and 4D scanning electron microscopy showed that lattice twisting occurred during the growth process, confirming that screw-dislocation-mediated layer-by-layer growth induced the chiral polyhedrons of the nanocrystals. That study was the first to demonstrate that screw-dislocation-mediated growth is the origin of the morphological chirality of the facets in single crystals.

### 2.7 Chiral biosensor and their biological effects

**Fig. 8:**
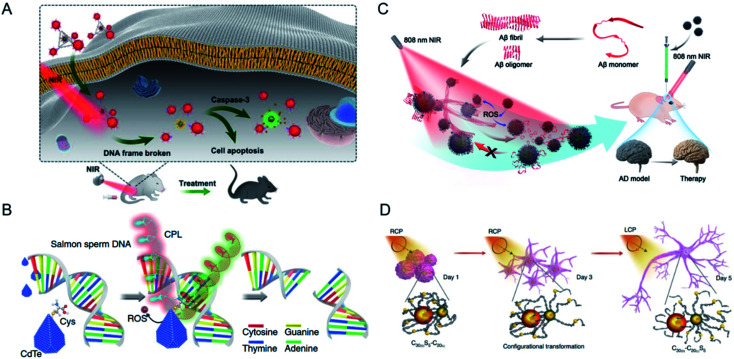
(A) Chiral tetrahedron assembly for NIR-induced clearance of senescent cells. Adapted with permission from ref. 98. Copyright © 2020 WILEY-VCH Verlag GmbH & Co. KGaA, Weinheim. (B) Schematic show the selectively cutting ability of chiral CdTe NPs for DNA under CPL illumination. Adapted with permission from ref. 101. Copyright © 2018 Springer Nature. (C) Chiral Fe_*x*_Cu_*y*_Se NPs for inhibition of Aβ42 monomer aggregation and enhancement of the disaggregation of Aβ42 fibrils under light. Adapted with permission from ref. 102. Copyright © 2020 Wiley-VCH Verlag GmbH & Co. KGaA, Weinheim. (D) Schematic of differentiation of NSCs under CPL illumination after incubation with chiral assembly for five days. Adapted with permission from ref. 107. Copyright © 2021 Springer Nature.

The research group of Xu and Kuang investigated the construction of chiral nanoprobes and their biological effects,^92–94^ and comprehensively studied the interactions between chiral nanomaterials and biosystems. An ultrasensitive detection method for biomolecules, including biomarkers of disease, biotoxins, bacteria, metal ions, and markers of cell metabolism, was established with self-assembled plasmonic chiral probes, based on their strong optical activity in the visible spectrum (Fig. 8A).^95–99^ The enhanced plasmonic CD signals in the visible range and the target-induced specific configurational changes in well-defined sensors resulted in much lower LODs or even allowed the detection of single molecules than other method. Dual signals produced by multifunctional imaging-based nanostructures were used to quantitatively detect cancer markers in both living cells and *in vivo*, extending the toolbox of chiral materials for the clinical diagnosis and treatment of cancer.^80,100^

Chiral NPs have been developed for the specific recognition of biomolecules, and have been shown to selectively cut both DNA and protein. Chiral nanomaterials have many features in common with natural chiral biomolecules. Therefore, the key purpose of our studies was to construct chiral nanostructures that not only mimic the functions of proteins, but also the scale matching with upstream or downstream metabolic pathways. Our initial studies have shown that after the appropriate chiral ligand (Cys) was identified, truncated tetrahedral chiral CdTe NPs with a diameter of 4.5 ± 0.3 nm cut salmon-sperm DNA under CPL illumination (Fig. 8B).^101^ Notably, these NPs sequence-specifically targeted the GATATC motif and site-selectively cut between adenine and thymine. The mechanism underlying this specificity of binding originated from the conformational match between the flexible targeted sequence and the truncated tetrahedral shape of CdTe. Based on this work, NPs with different chiral morphologies have been designed for use in biomolecule recognition. Recently, chiral Fe_*x*_Cu_*y*_Se NPs were fabricated with broad chiral optical activities, in the range of 400–1000 nm.^102^ These chiral NPs interfered with β-amyloid (Aβ) under 808 nm illumination, both *in vitro* and *in vivo* (Fig. 8C). The d-type NPs had much greater affinity for Aβ42 fibrils than the l-type NPs, and exerted a better therapeutic effect on neurodegenerative diseases. Copper(i) sulfide NPs were reported to recognize and cleave the core antigen of *Hepatitis B virus* (HBcAg), thus blocking viral transmission in living cells. Further confirmation was provided in a chronically infected transgenic mouse model, in which the therapeutic effect of the chiral NPs was better than that of a clinical drug. This illustrates the potential utility of chiral NPs as antiviral agents.^103^

Advances in this direction, clarifying the biological interactions between NPs of different handedness and living systems, have also been made. Chiral nanoassemblies modified with different glutathione (GSH) enantiomers have been used to regulate basic metabolic processes. Chirality-dependent d-GSH-coated structures displayed an enhanced autophagy-inducing ability, which was attributed to the different accumulation state of the structures. The degree of autophagy could also be quantified *in situ* from the intensity of the CD signal.^104^

A CPL-triggered mechanical force has also been identified in chiral assemblies.^105,106^ Based on the design of a DNA-based chiral assembly, CPL was used to stimulate the differentiation rate of neural stem cells into neurons (Fig. 8D). The chiral assembly became specifically entangled with the cytoskeletal fibers of the cells and an asymmetric force was generated under illumination, confirmed with both experimental and theoretical evidence, which deformed the actin in the cytoskeletons of the cells. The differentiated neurons were successfully implanted into the hippocampi of mice with Alzheimer's disease (AD), paving the way to utilizing the biological effects of CPL and chiral nanomaterials for biomedical applications.^107^

Notably, the research group of Tang and Liu showed that chiral gold NPs with a size of 3.3 nm had anti-Aβ therapeutic potential after they were coated with chiral GSH.^108^ A Monte Carlo simulation was used to show that the proper NP size was required to reduce the aggregation of Aβ42 peptide chains. The chiral NPs not only displayed chiral recognition to inhibit the aggregation of the Aβ42 peptide, a prominent histopathological marker of Alzheimer's disease, but also crossed the blood–brain barrier after their intravenous administration. The d-GSH-coated NPs inhibited Aβ42 fibrillization much more strongly than those coated with l-type GSH, by forming hydrogen bonds and electrostatic interactions with the peptides, both *in vitro* and *in vivo*. This study confirmed the potential utility of chiral nanomaterials as nanomedicines, based on their size- and stereo-determined recognition of peptides or proteins.

While exploring the chiral interactions between NPs and living systems to eliminate the size effect, the group of Robert Langer and Ana Jaklenec recently reported the chirality-dependent penetration efficiency of Co_3_O_4_ supraparticles (SPs) into living cells.^109^ In mechanistic studies, d-Cys-modified SPs showed much greater affinity for lipid bilayers than l-Cys-modified SPs, increasing their cellular internalization of the SPs. The stability of the d-SPs in mice was greater and their biological half-lives longer than those of l-SPs because the inert capping was not digested during metabolism. In a similar study of chiral Se NPs by Tianfeng Chen's group, a positron emission tomography (PET)-based method showed directly that l-GSH-modified Se NPs had a broader biodistribution in major organs than d- or dl-GSH-modified Se NPs. The researchers found that the homology between the NPs and the cytomembrane caused greater cellular uptake of the l-NPs, which then scavenged oxygen in living cells.^110^

## 3. Conclusions and perspectives

Although considerable advances have already been made in this area, further efforts are still required. First, a huge gap still exists in precisely defining the configurations of chiral NPs and molecules. Predictable stereoselective interactions between chiral materials and biomolecules will allow the biological processes of life to be controlled. The chiral recognition in metabolism, which is a fundamental process in the living body, is still an enormous challenge. Second, the development of much-more-sensitive detection methods for many biomolecules is still required. Stimulus-responsive chiral probes that can dynamically read variations in the biological environments of targets by themselves are particularly desirable. Chirality-based imaging devices for biosystems will also be extended to clinical applications.^111^ Third, the chiral effects of different nanomaterials are highly diverse. Current studies have shown that not only the surface properties, but also the size, scale, type, and even the state of nanomaterials influence the biological effects of nanostructures with different handedness. Comprehensive and fundamental investigations of the key factors that affect the behaviors of chiral nanomaterials in living cells are essential, and could unravel the mystery of the origin of chirality in life.

Further studies of chiral nanomaterials will build important bridges between these materials and living systems. The characteristics of chiral origins have inspired researchers in materials science to create chirality in various ways, even beyond natural architectures.

The exploration of chiral structures with extraordinarily high anisotropic factors, beyond those of plasmonic materials, is also warranted. A new and promising route to achieving this has emerged with the regulation of chiral properties by CPL or magnetic fields.^112–114^ Moreover, the specific recognition of biomolecules by chiral NPs has inspired us to create much more exquisite chiral structures to precisely modulate the performance of biological systems in living cells. Future research will mainly focus on the behaviors of chiral nanomaterials with other basic physiological functions, such as in immunity, metabolism, nutrition, and nerve conduction. The design of biodegradable and biocompatible properties in chiral NPs may further extend their application in the clinical context to chiral sensing, disease treatment, and antiviral defenses, such as against SARS-CoV-2, *Human immunodeficiency virus*, and HBV.

In summary, promising methods for the construction of chiral nanostructures with various building blocks have been described. As this field expands, there is no doubt that new technologies, new mechanisms, and new directions for designing chiral materials will be developed in the near future.

## Author contributions

Conceptualization: C. X.; writing-original draft: M. S., X. W., X. G., L. X. and H. K.; writing-review and editing: C. X.

## Conflicts of interest

The authors declare no competing financial interest.

## Supplementary Material
